# Differential Responses of Dinitrogen Fixation, Diazotrophic Cyanobacteria and Ammonia Oxidation Reveal a Potential Warming-Induced Imbalance of the N-Cycle in Biological Soil Crusts

**DOI:** 10.1371/journal.pone.0164932

**Published:** 2016-10-24

**Authors:** Xiaobing Zhou, Hilda Smith, Ana Giraldo Silva, Jayne Belnap, Ferran Garcia-Pichel

**Affiliations:** 1 Arizona State University, School of Life Sciences,Tempe, AZ 85287, United States of America; 2 Xinjiang Institute of Ecology and Geography, Key Laboratory of Biogeography and Bioresource in Arid Land, Chinese Academy of Sciences, Urumqi, Xinjiang 830011, China; 3 U. S. Geological Survey, Southwest Biological Science Center, Moab, UT 84532, United States of America; 4 Center for Fundamental and Applied Microbiomics, Biodesign Institute, Arizona State University, Tempe, AZ 85287, United States of America; University of Milan, ITALY

## Abstract

N_2_ fixation and ammonia oxidation (AO) are the two most important processes in the nitrogen (N) cycle of biological soil crusts (BSCs). We studied the short-term response of acetylene reduction assay (ARA) rates, an indicator of potential N_2_ fixation, and AO rates to temperature (T, -5°C to 35°C) in BSC of different successional stages along the BSC ecological succession and geographic origin (hot Chihuahuan and cooler Great Basin deserts). ARA in all BSCs increased with T until saturation occurred between 15 and 20°C, and declined at 30–35°C. Culture studies using cyanobacteria isolated from these crusts indicated that the saturating effect was traceable to their inability to grow well diazotrophically within the high temperature range. Below saturation, temperature response was exponential, with Q_10_ significantly different in the two areas (~ 5 for Great Basin BSCs; 2–3 for Chihuahuan BSCs), but similar between the two successional stages. However, in contrast to ARA, AO showed a steady increase to 30–35°C in Great Basin, and Chihuhuan BSCs showed no inhibition at any tested temperature. The T response of AO also differed significantly between Great Basin (Q_10_ of 4.5–4.8) and Chihuahuan (Q_10_ of 2.4–2.6) BSCs, but not between successional stages. Response of ARA rates to T did not differ from that of AO in either desert. Thus, while both processes scaled to T in unison until 20°C, they separated to an increasing degree at higher temperature. As future warming is likely to occur in the regions where BSCs are often the dominant living cover, this predicted decoupling is expected to result in higher proportion of nitrates in soil relative to ammonium. As nitrate is more easily lost as leachate or to be reduced to gaseous forms, this could mean a depletion of soil N over large landscapes globally.

## Introduction

Biological soil crusts (BSCs) are complex soil surface communities with cyanobacteria, green algae, lichens or mosses as primary producers [1]. They also contain significant populations of heterotrophic bacteria, archaea [2] and fungi [3], as well as chemolithotrophic microbes [4]. They are common in dryland regions, where they can reach up to 70% of the living cover [5], and thus are an important element of these ecosystems both locally and globally [6]. Such communities have colonized soil surfaces at least since the Proterozoic and represent the only known terrestrial ecosystem at that time [7]. Cryptogamic covers around the world, much of which are BSCs, can fix as much as 49 Tg of nitrogen (N) per year, which accounts for nearly half of the global biological N_2_ fixation on land [8]. Biological soil crusts affect most dryland ecosystem processes. They stabilize the soil surface [9, 10], fix atmospheric carbon (C) and N [8, 11–15], alter the soil content of many metals and metalloids [16], may help maintain vascular plant diversity [17, 18], and affect hydrological processes [19–21].

Dinitrogen fixation is also the entry point of an active N cycle in BSCs. Most N_2_ fixation in BSCs is done by heterocystous cyanobacteria (either free-living or in association with lichens). Surveys of *nifH* genes have revealed that three generic entities (*Nostoc* spp., *Scytonema* spp. and *Tolypothrix/Spirirestis* spp.) are dominant in BSCs [22], but a few heterotrophic bacteria are also important in early successional BSCs that are devoid of heterocystous cyanobacteria [23]. Variable rates in time and space and with BSC successional stage have been documented in a wealth of studies [12, 14, 15, 24, 25]. Within a given BSC type, moisture and temperature (T) are the two most important abiotic factors that determine N_2_ fixation rates [12, 26, 27].

Ammonia oxidation (AO) behaves as the crucial step to transform the fixed, reduced N to nitrate (NO_3_^-^). It is the first and rate-limiting step of nitrification, with chemolithoautotrophic *Proteobacteria* and archaea in the phylum *Thaumarchaeota* conducting this process in soils and BSCs [2, 28]. The AO bacteria and archaea seem to differ in their relative importance with changes in factors such as moisture, pH, and fertilization treatments [29–32]. Ammonia oxidation is a prominent transformation in BSCs across biogeographic regions in the Southwestern USA [14] and seasons [33], usually showing rates similar in magnitude to those of N_2_ fixation [14, 33].

Denitrification indicates the reduction of nitrates to gaseous N_2_ by microorganisms. In North American soil BSCs, denitrification and anaerobic ammonia oxidation (anammox), whenever measured concurrently, show orders-of -magnitude rates lower than N_2_ fixation and AO [14, 33]. It is not known why rates of denitrification and anammox are so low, although it is possibly due to low denitrifier population or insufficient NH_4_^+^ and NO_3_^-^to act as substrates [14, 34]. Thus, the balance between AO and N_2_ fixation rates largely determines the net N budget in BSCs.

Nitrogen cycling in microbial communities of drylands are structured by abiotic factors to a stronger degree than what is generally observed in more mesic systems [35]. Temperature is one of chief factors that affect all aspects of the N cycle, including N_2_ fixation and AO. In drylands, T varies greatly within a day and among seasons and years. Indeed, T changes (between 5°C and 30°C) can be more important than variation in soil water content (between 30% and 80% of field capacity) in determining the relative dominance of N transformation rates in BSCs from a semi-arid grassland [36]. In BSCs, T can also shape microbial community composition, including those of N_2_ fixing cyanobacteria [37] and AO microbes, selecting for AO archaea in warmer deserts and for AO bacteria in cooler deserts [35]. And yet, many studies that have simultaneously compared multiple transformations in the N cycle have not directly addressed the potential effects of T on interactions among different processes. Because these effects do not need to scale equally for different N transformations, T variations may decouple these processes, producing divergent net outcomes.

In this study, we describe the T sensitivity of both the acetylene reduction assay (ARA), as an indicator of N_2_ fixation, and AO, on BSCs using short-term acclimation experiments. In order to gauge potential effects of long-term acclimation to different T regimes, we included BSCs from a cool (Great Basin) and hot (Chihuahuan) desert. The BSCs were incubated under different T treatments with sufficient moisture supply to explore the sensitivities of both ARA and AO to site-relevant T variations, and also to ascertain potentially differential responses to BSCs from different locations under current and future Ts. We hypothesized that rates of ARA and AO would display optimal Ts that would be relatively higher in hot deserts compared with cool deserts, due to long-term adaption of their respective microbial populations, and sought to establish if, by virtue of having differential effects on different processes, future changes in T may change the balance in dryland N cycles.

## Materials and Methods

### Site descriptions and sample collection

Our samples were obtained from Hill Air Force Base in the Great Basin Desert (USA) (41.10° N, 113.00° W) and from the Jornada Experimental Range in the Chihuahuan Desert (USA) (32.55° N, 106.72° W), which are typical of cool and hot desert ecosystems, respectively. At Hill, mean annual precipitation is 200 mm and mean air T is 3°C in January and 34°C in July. At the Jornada, the mean annual precipitation is 280 mm and the mean air T is 14°C in January and 36°C in July.

Intact BSCs were collected to 1.5 cm depth with a putty knife in October of 2014 in the Great Basin and in March 2015 in the Chihuhuan Desert, respectively. The patches of intact BSC were randomly collected at each site and transported to Arizona State University in wooden frames that preserved their integrity. Samples were kept dry (inactive) under room temperature (around 25°C) and dark until the start of the laboratory experiment. The BSCs in the field was extremely dry so that the microbial activity was very low, and humidity in the storage unit was also kept low [38]. The experiment was started within days of each sampling. Visual inspection (such as color and surface roughness determination) is an indicator of rough BSCs species composition and biomass, and was used in our study to describe our BSC types. We used early successional (light; dominated by motile *Microcoleus*-like cyanobacteria and late successional crusts from each site (dark or lichen crusts), according to the naturally occurring climax community [39].

In the Great Basin soils were silty clays. In the Chihuahuan Desert, light and lichen BSCs were used. The two BSCs in the Chihuahuan desert were on sandy soil. Light BSCs refers to the light coloration of the soil surface, and are dominated by early successional, motile cyanobacterial species [39]. Dark BSCs refer to the darker color imparted to the soil surface by heavily pigmented non-motile cyanobacteria and develop secondarily from light crust in a later successional stage [1]. Lichen BSCs are typically a late successional crust type, where the surface is dominated by lichens [40].

### Soil parameters

Pigment, geochemical parameters, and pH were determined and three replicates were included for each measurement. For pigment concentrations, 2 cm^2^ of collected BSC sample was ground, put into a 15 ml falcon tube, and mixed with 10 ml 90% acetone. The mixture was kept in dark at 4°C for 24 h, and then centrifuged at 3000 *× g* for 10 min. Supernatant absorbance was read at 384nm, 490 nm, and 663nm. Chlorophyll *a* and scytonemin contents were calculated based on Garcia-Pichel and Castenholz [41]. Soil geochemical parameters (total carbon (C), total N (N), NH_4_^+^-N and NO_3_^-^-N) for each sample were determined commercially at the Core Research Facilities Administration, Arizona State University. Ten g fresh soil samples were exacted with 50 ml 2M KCl solution, and NH_4_^+^-N and NO_3_^-^-N were analyzed by a AQ2 Discrete Analyzer system (SEAL Analytical, Inc., WI, U.S.A.). For total C and N, air-dried soil samples were ground in a ball mill (SPEX SamplePrep, NJ, U.S.A.) and analyzed on a PE2400 Elemental Analyzer (PerkinElmer, Inc, MA, U.S.A.). Soil solution pH was measured with a 1: 5(W/V) of BSCs sample: double-dionized water that was shaken for 3 min, allowed for equilibrate for 24 h, and determined with a pH meter.

### DNA exaction and real-time PCR

Sample DNA was extracted from 0.4 g BSCs using the PowerSoil DNA isolation Kit (MO BIO Laboratories, Inc. USA), and quantified using the SYBR Green assay as described by Brankatschk et al. [42]. SYBR green based real-time PCR assays were run on an ABI7900HT thermocycler (Applied Biosystems, Foster City, CA). Reaction volume was 20 μL and iTaq SYBR GREEN Fast PCRmaster mix. PCR primer for N_2_ fixation gene were *nifH*F (AAAGGYGGWATCGGYAARTCCACCAC) and *nifH*R (TTGTTSGCSGCRTACATSGCCATCAT), for *amoA* were *amoA*1f mod (GGGGHTTYTACTGGTGGT) and *AmoA*-2R’(CCTCKGSAAAGC- CTTCTTC). Each PCR run included triplicate sample templates, calibration standards and no-template control.

### Temperature pre-treatments

One cm deep, intact BSCs pieces were cut and modified carefully with cutter blade from the patch of the field sampled BSCs and then placed into the wells of a six well (diameter = 3.5 cm) tissue culture plate. One well was reserved for autoclaved samples (controls) and five for treatment samples. Each well contained around 10 g of BSC material. Double deionized water was filled until the soil reached its water holding capacity (a thin film of water remained on the sample surface). The plates were covered and incubated at different Ts in separate incubators, all illuminated at a light intensity of around 80 μmol (photons) m^-2^ s^-1^. Analyzed Ts were -5, 5, 10, 15, 20, 25, 30 and 35°C. The levels of illumination roughly correspond to that of a heavily overcast, rainy morning. The temperature ranges were selected to encompass the variability in air temperature changes around the two sites. During the whole incubation period, the samples were kept intact. Water loss in the well was slow during the incubation and soil water content was stable. The pre-incubation time was 24 h, and the incubated samples were used for the measurements of potential N_2_ fixation rates and AO rates, each at their respective T. Eight independent replicates were used in potential N_2_ fixation and five for AO rates measurements, because ARA values are often more variable.

### Potential N_2_ fixation rates

Potential N_2_ fixation rates were estimated using ARA according to the methods of Belnap [12]. The samples for incubation were cut out of the collected intact material using clear, gas–tight tubes (diameter of 2.54 cm, length of 9.5 cm), put in the tubes, and closed by rubber stoppers with the top end having septum ports for sampling (eight replicates for each treatment). Tubes were injected with acetylene (C_2_H_2_) to create a 10% C_2_H_2_ atmosphere and incubated for 5 h at the various Ts. Gas (4 ml) of the headspace within the tubes was collected and analyzed for C_2_H_2_ and ethylene (C_2_H_4_) content. The samples were analyzed on a Shimadzu GC-14 A gas chromatograph, using helium as the carrier gas (30 ml min^-1^). Calibrations with ethylene standards were done at the time of observations. Results of the observed nitrogenase activity as nmol C_2_H_4_ m^-2^ h^-1^ were transformed to N input (μmol N m^-2^ h^-1^) using the theoretical conversion ratio 3 [26].

### Potential AO rates

Potential (ammonium amended) aerobic AO rates were determined according to the methods described by Strauss et al. [14] and Maruseko [35]. A mixture of 20 mM sodium chlorate (NaClO_3_, an inhibitor of nitrite reduction) and 1mM ammonium sulfate ((NH_4_)_2_SO_4_) was prepared and adjusted to pH 7.2 using KH_2_PO_4_ and K_2_HPO_4_. The samples, which had been pre-incubated under different Ts, were slurried with this mixture (1:4 w/v) (five replicates for each treatment). These samples were incubated for 6 h in the dark in an environmental shaker, set at the corresponding pre-incubation T. Aliquots of 5 ml were removed every 3 h and mixed with 5 ml 4M KCl. Samples were centrifuged at 5,000 *×* g for 5 min to pellet the debris. The supernatant was filtered with Whatman #42 filters and then nitrite (NO_2_^-^) was determined spectrophotometrically at 520 nm, after color reagent reactions. NO_2_^-^ production was calculated as the linear increase in NO_2_^-^ concentrations over time (μmol N m^-2^ h^-1^).

### Cyanobacterial cultures isolation, growth and thermophysiology

We isolated the N_2_-fixing cyanobacteria, *Nostoc* spp., *Tolypothrix* spp. and *Scytonema* spp., from each of the field locations by using enrichment cultures in liquid media where N_2_ was the only N source (B11_0_.; see Yeager et al. [22]). Resulting colonies were then streaked on 1.5% agar plates and observed with optical microscopy to corroborate the presence of only one of the desired morphotypes per culture (unialgal). For each culture the 16S rRNA gene was sequenced using cyanobacterial specific primers [43], to corroborate identity by blasting against GenBank using BLASTN [44]. Cultures are maintained in the culture collection of the Garcia Pichel’s laboratory at ASU and are publicly available upon request. To evaluate responses to T under N_2_-fixing conditions, strains were inoculated in 20 mL of N free media (B11_0_) at 5% v/v in 50 mL culture bottles. The cultures were incubated at different T (15, 25, 30, 35 and 40°C) in a 12 h photoperiod, and illuminated for 30 days at 20–27 μmol m^-2^ s^-1^. Growth was estimated visually in three categories: optimal, suboptimal and no-growth, which included dying cultures. The experiment was replicated in full, and growth in either one of the trials reported as a positive.

### Statistics

For each type of BSC, one-way ANOVA was used to determine the differences in ARA and AO rates among the different T treatments, and LSD method was applied for multi-comparisons. Normality and homoscedasticity were tested before ANOVA analysis. Within each desert, t-test statistics was performed on the two indices between light and dark/lichen BSCs at each T. The statistical analysis were conducted using SAS software (Version 8.0, SAS Institute Inc., Cary, NC, U.S.A.) at the α = 0.05 level. The Arrhenius equation, which displays the logarithm of kinetic constants versus inverse T, was used to gauge the effect of T on the rates of N transformation. Only the portion of each dataset where the rates increased with T was used so as to explicitly avoid T ranges where process rates were saturated. The form of the equation can be written as ln (K) = ln (β)+ α(1/T), where K is rate, T is absolute T in degrees Kelvin, and ln (β) is the value of the true y-intercept (1/T = 0) and α is the slope of the regression line. The relationship between ln (k) and 1/T was determined with reduced major axis (RMA) regression. The slope α and ln (β) were obtained using standardized major axis (SMA) regression SMATR software package protocols. The software package was also used for post-hoc multiple comparison of slopes among the eight different groups. The Q_10_, which indicates the average fold increase in rate for an increase of 10°C, was also calculated from exponential regressions of the same datasets. Significance was defined as *P* < 0.05.

## Results

### Biological soil crust characterization

The main characteristics of the BSC used are presented in Table 1. As intended, differences in BSC successional stage (maturity) were reflected in the proxy of phototrophic biomass (chlorophyll *a*), which for a given locality were lower in light than in dark/lichen BSCs. Samples from the Great Basin had significantly more chlorophyll *a* than those of Chihuahuan Desert (*P* < 0.05), regardless of successional stage. More mature BSCs had a higher concentration of scytonemin, reflective of larger numbers of heterocystous cyanobacteria [45]. Soil pH was moderately alkaline in all BSCs, and slightly, but significantly, lower in samples from the Chihuahuan than those from the Great Basin deserts. The abundance of the functional genes *amoA* and *nifH* and NH_4_-N concentrations was higher in later than earlier successional BSCs in both deserts. The abundance of *amoA* and *nifH* in early successional BSCs was similar in both deserts; however, the abundance of *amoA* and *nifH* in late successional BSCs from the Great Basin were 5.1 and 2.0 times higher, respectively, than those in late successional BSCs from the Chihuahuan desert. The BSCs from the Great Basin had relatively higher concentrations of NH_4_-N and NO_3_-N than those in the Chihuahuan desert.

**Table 1:** Pigment concentrations and soil chemistry and gene abundance of *nifH* and *amoA* in different BSCs types of the Great Basin and Chihuahuan Desert (mean ± s.e., n = 3).

		pH	Chl *a*	Scytonemin	TN	TC	NH_4_-N	NO_3_-N	*nifH*	*amoA*(AOB)
Origin	Type		μg/cm^2^	μg/cm^2^	(%)	(%)	(μg/g)	(μg/g)	copies g^-1^	copies g^-1^
Great Basin	Dark	8.12±0.08a	3.15±0.09 a	47.03±1.10 a	0.18 ±0.00 a	4.92 ±0.17 a	9.54±0.67a	1.79±0.09b	2.63×10^13^a	1.99×10^12^ a
Great Basin	Light	8.12±0.11ab	2.41±0.32 b	5.00±0.24 c	0.17 ±0.02 a	4.86 ±0.14 a	7.85±0.41b	3.03±0.33a	3.94×10^12^b	7.43×10^11^ b
Chihuahuan Desert	Lichen	7.94±0.02ab	1.81±0.22 bc	32.17±2.10 b	0.09 ±0.01 b	2.12 ±0.06 b	7.6±0.40b	0.94±0.07c	5.13×10^12^b	9.80×10^11^ b
Chihuahuan Desert	Light	7.91±0.02b	1.24±0.15 c	2.30±0.23 c	0.04 ±0.00 c	1.08 ±0.16 c	4.08±0.23c	0.67±0.06c	2.52×10^12^b	5.92×10^11^ b

Note: TN = total nitrogen, TC = total carbon, Chl *a* = chlorophyll *a*.

### Acetylene reduction rates

**Fig 1:**
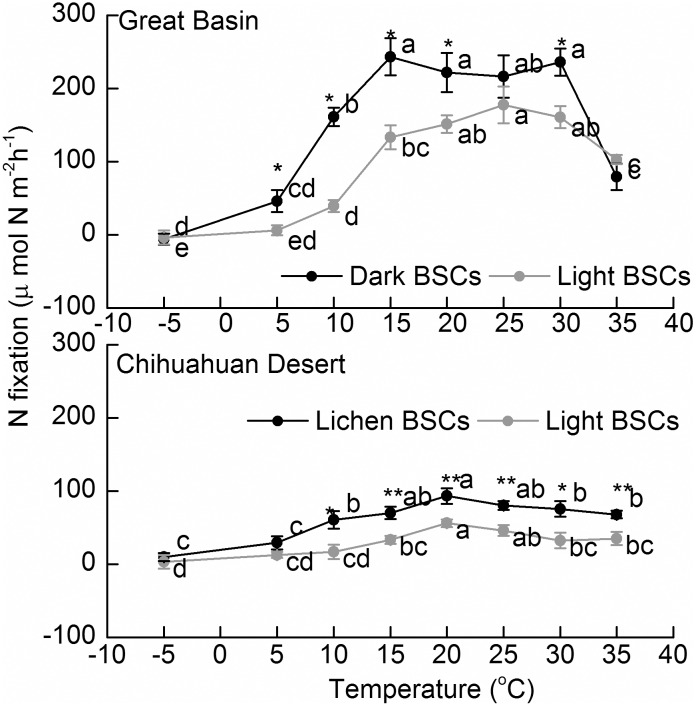
Nitrogen fixation (Acetylene reduction) rates of dark (solid black symbols), light (grey symbols) and lichen (solid black symbols) BSCs in the Great Basin and Chihuahuan Desert. Symbols show the mean of n = 8 determinations and error bars depict standard errors. Different lowercase letters indicated significant differences (p < 0.05) for comparisons among temperatures within single BSC type. An asterisk indicates significant differences between dark and light BSCs for a single temperature.

We found similar trends with T of potential N_2_ fixation rates for light, dark and lichen BSCs from either desert (Fig 1). In all BSCs, rates increased with increasing T until reaching an optimal range where they leveled off, which was reached between 15 and 20°C. The plateau in optimal T ranged between 15 and 30°C, with ARA declining markedly at 35°C, in the Great Basin samples. In the Chihuahuan, optima plateaus were around 15–25°C and above that there was a slight but significant decrease. Rates were significantly higher in dark/lichen compared to light successional BSCs in both deserts at most Ts. Rates in the Great Basin BSCs were much higher than those in the samples from the Chihuahuan. Below the optimum range, the sensitivity of ARA (as judged by Q_10_ or the slope of Arrhenius plots on Table 2 and S1 Fig) was not significantly different between the two BSC types from the same region, but BSCs from the northern Great Basin had a much stronger response to T than Chihuahuan Desert BSCs.

**Table 2:** Responsivity of N transformations to T among the BSCs studied, gauged by estimates of the slope of a regression between the natural logarithm of the process rates and the inverse of temperature (Arrhenius plots; see full data in S1 Fig). Only the portion of any dataset where rates increased with T was used. Slopes that were not significantly different at the 95% were assigned same letter in the multi-comparison column. Q_10_s, giving the average fold increase in rate for an increase of 10°C, are also included, calculated from regression curves of the same datasets.

Origin	Type	Process	n	R^2^	Slope	Multi-comparison (95% significance)	Q_10_
Great Basin Desert	Dark	ARA	3	0.93	-13380	b	5.2
		AO	6	0.92	-12712	b	4.5
	Light	ARA	5	0.83	-13481	b	5.0
		AO	6	0.96	-13290	b	4.8
Chihuahuan Desert	Lichen	ARA	4	0.96	-7231	a	2.5
		AO	7	0.96	-7632	a	2.4
	Light	ARA	5	0.99	-8757	a	3.1
		AO	7	0.93	-7996	a	2.6

Note: ARA = acetylene reduction rate, AO = ammonia oxidation rate.

### Potential AO rates

**Fig 2:**
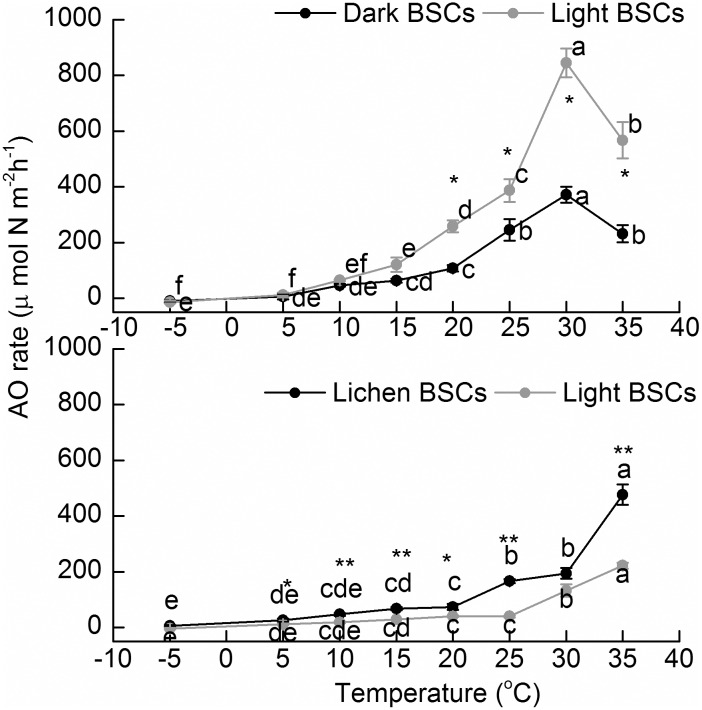
Potential AO fixation rates of dark (solid black symbols) and light (grey symbols) and lichen (solid black symbols) BSCs in the Great Basin and Chihuahuan deserts. Symbols show the mean of n = 5 determinations, anderror bars depict standard errors. Different lowercase letters indicated significant differences (p < 0.05) for comparisons among temperatures within a single BSC type. An asterisk indicates significant differences between dark/lichen and light BSCs for a single temperature.

The potential AO rates of the BSCs in the two deserts also increased with T, but did not show a wide plateau of close-to-optimal Ts such as those found for potential N_2_ fixation (Fig 2). Chihuahuan Desert samples increased steadily within the ranges tested; Great Basin Desert samples suffered losses at the highest T. Similar to ARA, the sensitivity of AO to T, as gauged by Q_10_ or the slopes of Arrhenius plots (S1 Fig, Table 2), was similar among BSC types in a given desert, but much higher in BSCs from the Great Basin than in those from the Chihuahuan Desert.

### Growth of N-fixing cyanobacteria as a function of T

All 30 strains of diazotrophic cyanobacteria showed robust diazotrophic growth at 15 and 25°C, regardless of their origin (Table 3). However, clear differences were seen at high Ts. No *Nostoc* spp. Cultures grew well at 30°C or above, and only 4 out of 8 strains of *Tolypothrix* spp. did, but all strains of *Scytonema* spp. grew well. At 35 and 40°C, from all strains of all taxa, only 11 out of 12 *Scytonema* spp. strains grew.

**Table 3:** Diazotrophic growth capability as a function of temperature in cultured cyanobacteria isolated from the Great Basin and the Chihuahuan deserts.

Taxon	Number of strains tested				Number of strains growing at				
	Great Basin (Cold)		Chihuahuan (Hot)		15°C	25°C	30°C	35°C	40°C
	Light	Dark	Light	Dark					
*Nostoc* spp.	2	4	3	1	10	10	0	0	0
*Tolypothirx* spp.	4	2	1	1	8	8	4^a^	0	0
*Scytonema* spp.	1	4	2	5	12	12	12	11^b^	11^b^

^a^ Four strains of *Tolypothrix* spp. did not grow at 30°C, three were isolated from light crust (two from the cold and one from the hot desert), and one was isolated from light crust in the cold desert.

^b^ One strain of *Scytonema* spp. did not grow at 35 and 40°C. This strain was isolated from light crust in the hot desert.

## Discussion

### Temperature influence on the N transformations of biological soil crusts

We found a similar pattern of N_2_ fixation in the four BSC types/origins: ARA increased with T until reaching a wide optimal plateau, after which it decreased. The plateau varied somewhat with locale and maturity, around the range 15–30°C. Most studies have found a similar pattern and optimal ARA rates at this T range, regardless of BSC type or location. In the vicinity of Moab, UT, Barger et al. [26] found that both light and dark BSCs from cool deserts (Canyonlands National Park, USA) had peak rates in the range of 20 to 30°C. In the Tenneger Desert, China, Su et al. [15] found an optimal range of ARA at 15–30°C. Global literature reviews that include studies from the Arctic, Antarctica, Scotland, Canada, South Africa, and subalpine regions also found the optimal T range of 20–30°C [26, 46]. Because similar optimal ranges have been observed across so many soil types and environmental conditions, it could represent basic 1) thermal, 2) nutrient, or 3) energy constraints on the few dominant organisms generally associated with N_2_ fixation in BSCs (largely the cyanobacteria *Nostoc* spp., *Scytonema* spp. and *Tolypothrix/Spirirestis* spp. [22]*;* although some heterotrophic diazotrophs do help when those cyanobacteria are absent [23]). To compare this optimal range for N_2_ fixation with the T dependence of their diazotrophic metabolism, we carried out growth experiments with representative isolates of each of the major cyanobacterial diazothrophs (Table 3). It is clear from this data that a large proportion of the strains cannot continue growing at T above 30°C, and that the genera that dominate in Great Basin crusts (*Tolypothrix* spp. and *Nostoc* spp. [37]) are much more sensitive than those that are more common in the Chihuahuan, warmer crusts (*Scytonema* spp. [37]). Thus the phenomenology of N_2_-fixation (Fig 1) can likely be traced to the community composition and physiology of their inhabiting diazotrophic flora.

The patters in the crusts are also consistent with nitrogenase degradation above 39°C [47, 48]. Zhu and Brill [49] and Brooks et al. [47, 49] also both found that regulatory proteins coded by *nifA* were suppressed at 37–41°C. In general terms, the T dynamics of N_2_ fixation are similar to those found for net photosynthesis in BSCs [50, 51]. It is possible that the effects seen in N_2_ fixation are a consequence of a limited C or ATP supply, both in the field and in our cultures. Regardless of the mechanisms behind the observed decline in ARA at higher T, it has implications for scaling of T with other processes involved in the BSC N cycle, as is discussed below.

Interestingly, while there were no major differences in the optimal ranges for the BSCs between the cool and hot desert tested, the samples in the cool Great Basin did show a much more precipitous decrease at high T, probably due to the low incidence of *Scytonema* spp. cyanobacteria there. In this dual sense our results are only partially consistent with our initial hypothesis that ARA rates would display relatively higher optimal Ts in hot compared with cool deserts. This implies that the N fixing cyanobacteria present in these deserts are not just being selected for their ability to fix N under normal thermal regimes. Much clear effects can be seen from the literature: optimal T for ARA is 35–40°C for cyanobacterial BSCs s in the cool Loess Plateau region of China [27], but only 25°C in BSCs from the High Arctic [52]. Our ARA rate did, however, show significant (Table 2) and consistent differences at the low end of the T range: late successional BSCs from the cooler Great Basin showed a much more marked response to T than those from the hot desert, with Q_10_ values almost twice as large within below 20°C, indicating that the extant populations in those samples can make better use of “warm spells” or seasons. A Q_10_ value measures the T sensitivity of an enzymatic reaction rate or physiological process with a T increase of 10°C. Most biological systems show Q_10_ values about 2–3 [53], but they can vary significantly between processes, even in a single system. For instance, between 25 and 15°C, Q_10_ values in the H_2_-evolving symbioses of *Rhizobium japonicum* ranged from 2.0 to 2.7 for CO_2_ evolution, 1.3 to 2.4 for C_2_H_2_ reduction, and 3.2 to 3.7 for H_2_ evolution [54].

In the case of potential AO, we did not observe a wide plateau of optimal rates. The relatively higher optimal T for BSCs from the Chihuahuan than from Great Basin was consistent with our initial hypothesis that AO rates would be higher in hot desert compared to cool desert soils. The relationship of AO rates was clearly exponential with T for most of the T range we tested (Fig 2; Table 2), thus behaving much like a purely chemical reaction and fitting well a log-linear relationship. In the present study, only samples from the Great Basin showed AO inhibition at higher Ts (a symptom of the organismal or biochemical machinery failing), which is consistent with an adaptation of the ammonia oxidizers to the local T regime, as would be anticipated based on past molecular studies [35].

### Balance between N_2_ fixation and AO as influenced by temperature

We found consistent and significant trends in T response of ARA and AO. The Arrhenius slopes and Q_10_‘s of the Chihuahuan Desert samples (Q_10_ = 2.50–3.06) were roughly half of the Great Basin Desert samples (Q_10_ = 4.80–5.15), an indication that ammonia oxidizers, like N fixers in the cool deserts are much less T-dependent that those found in the hot desert samples and unusually responsive to T. The reasons behind this are unclear to us.

**Fig 3:**
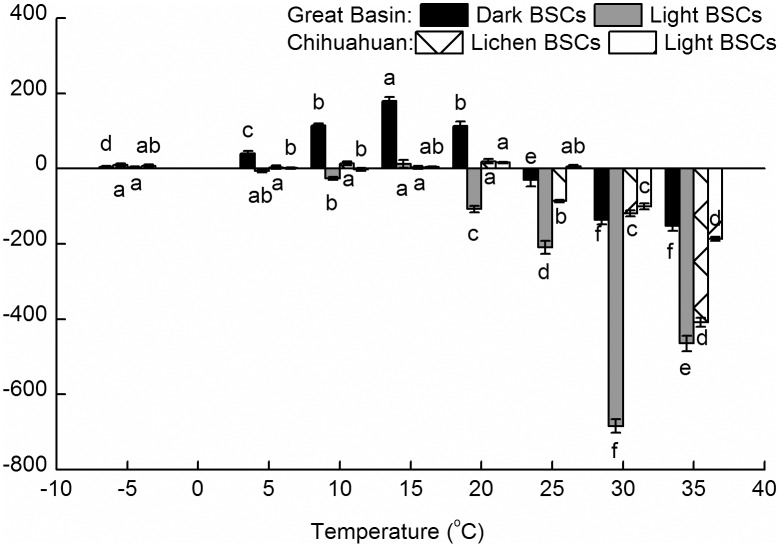
Trend of the balance between Nitrogen fixation (acetylene reduction) and AO rates, as the net difference for each BSC type an origin, according to the legend inserted. Columns show the mean of n = 40 determinations (differences between 8 ARA value and 5 AO value), and error bars depict standard errors. Different lowercase letters indicated significant differences (p < 0.05) for comparisons among temperatures within a single BSC type.

The major differential effect of T on ARA and AO was the presence and absence, respectively, of a ‘saturation’ or plateau at the mid to warmer end of the T range, rather than a differential response to T at the lower end (in fact the Q_10_ values for both processes at lower Ts were not significantly different in either geographic area; Table 2), regardless of geography or BSC type. This resulted in an imbalance between ARA and AO at warmer Ts that was not documented at lower Ts (Fig 3). However, it is important to note that our experiments could only assess potential N_2_ fixation and AO. Therefore, the absolute values and sign of the imbalance may not be accurate. In fact, it is likely that actual AO rates are somewhat overestimated and N_2_fixation rates somewhat underestimated in our assays [33] because of artificially increased diffusion of oxygen in the AO assay, and artificially increased diffusion of acetylene/ethane in the ARA assay. Thus, our results should be used for assessment of relative changes. Despite this limitation, we see a strong and consistent pattern of AO becoming relatively more important at T above 15–20°C, as ARA (indicating N inputs) stay constant or decline. Because the denitrification rates in the two deserts are at least an order of magnitude smaller than N_2_ fixation [14], we therefore predict that as drylands warm, the relative proportion of soil N will shift away from ammonium and towards nitrate, which is more easily lost via leaching, resulting in less overall N.

While experiments examining warming effects on soils crusts do exist, none have focused directly on gauging effects on the N cycle. A small increase of 2°C did not seem to affect soil chemistry in the short term [55, 56]. In another study where warming treatments were applied in the field, the warming treatment decreased levels of available N [36], although it is not known whether this was a result of the concomitant decline in BSC cover or because AO increased during times of declining N_2_ fixation, as we observe.

## Conclusions and Outlook

As global temperatures rise in the future, many ecosystem processes will likely be altered. Our findings suggest that N cycles may be one process that is significantly affected. Many studies have documented that as higher temperatures become more common, N inputs via fixation are likely to decrease. Our results show that in addition to N inputs declining with rising temperatures, AO rates will likely continue to rise, thus decoupling the input and transformation processes. If our laboratory experiments can be extrapolated to the field, we predict this decoupling will result in a higher nitrate to ammonium ratio in dryland soils. Because nitrate is more easily lost both through leaching and as a gas than ammonium, we also expect an overall reduction in soil available N. As most desert soils are already N-limited, and cover ~35% of the Earth’s surface, the decoupling observed in this study could have large implications for soil fertility and thus plant productivity at the global scale. For example, a recent study by Poulter et al.[57] in Nature found that ~60% of global C sink anomalies were driven by semi-arid vegetation growth in Australia alone. Because of the relevance of BSCs to N budgets across all scales [8, 12], and the subsequent effect of N limitation on C cycling [58], we need long-term field experimentation to test the validity of our prediction. This is especially important in the face of the multiplicity of factors that may affect the N balance under natural conditions (e.g., soil moisture, CO_2_), many of which will likely be affected by future warming.

## Supporting Information

S1 Fig Linear regression between ln(N_2_ fixation rate) (A) and ln (AO rate) (B) with 1/T based on the Arrenius equation. GB-Dark: Dark BSCs of the Great Basin; GB-Light: Light BSCs of the Great Basin; CH-Lichen: Lichen BSCs of the Chihuahuan Desert; CH-Light: Light BSCs of the Chihuahuan Desert. Only the portion of dataset where the rates increase with T was used.
(DOCX)
