# Supplementary material for: Differential Responses of Dinitrogen Fixation, Diazotrophic Cyanobacteria and Ammonia Oxidation Reveal a Potential Warming-Induced Imbalance of the N-Cycle in Biological Soil Crusts
Source: PLoS One. 2016 Oct 24;11(10):e0164932. doi: 10.1371/journal.pone.0164932 (PMC5077114; doi:10.1371/journal.pone.0164932)
Supplement: S1 Fig — GB-Dark: Dark BSCs of the Great Basin; GB-Light: Light BSCs of the Great Basin; CH-Lichen: Lichen BSCs of the Chihuahuan Desert; CH-Light: Light BSCs of the Chihuahuan Desert. Only the portion of dataset where the rates increase with T was used. (DOCX) [file pone.0164932.s001.docx]

**

**

**S1 Fig.** Linear regression between ln(N_2_ fixation rate) (A) and ln (AO rate) (B) with 1/T based on the Arrenius equation. GB-Dark: Dark BSCs of the Great Basin; GB-Light: Light BSCs of the Great Basin; CH-Lichen: Lichen BSCs of the Chihuahuan Desert; CH-Light: Light BSCs of the Chihuahuan Desert. Only the portion of dataset where the rates increase with T was used.
